# Substantial decrease in paediatric lower extremity fracture rates in German hospitals in 2017 compared with 2002: an epidemiological study

**DOI:** 10.1186/s12891-020-03393-0

**Published:** 2020-06-08

**Authors:** Christoph Emanuel Gonser, Christian Bahrs, Philipp Hemmann, Daniel Körner

**Affiliations:** Department of Traumatology and Reconstructive Surgery, BG Trauma Centre Tübingen, Eberhard Karls University Tübingen, Schnarrenbergstr. 95, 72076 Tübingen, Germany

## Abstract

**Background:**

There are no recent studies on the frequency of paediatric lower extremity fractures in Germany. The aims of this study were to report fracture rates of paediatric lower extremity fractures treated in German hospitals in 2002 and 2017 and to detect changes over time as well as to evaluate the gender and age distribution for each fracture location.

**Methods:**

Data from the German National Hospital Discharge Registry, which covers over 99% of all German hospitals, were used for this study. The absolute frequencies and incidence of lower extremity fractures as well as age at the time of fracture and gender were included in the data. The population was subdivided into four age groups: 0–4, 5–9, 10–14, and 15–19 years. The boy: girl ratio (BGR) for all fracture locations was calculated by dividing the absolute frequency of boys by that of girls. The fracture incidence in 2017 was compared with 2002 by calculating the incidence rate ratio (IRR).

**Results:**

The total number of fractures decreased by 39.9% from 2002 to 2017. The most common fracture locations in 2002 were femoral shaft, tibial shaft, distal tibia, and lateral and medial malleolus; the absolute number of all these fractures was lower in 2017 than in 2002 in all age groups. The incidence of hip and thigh fractures, knee and lower leg fractures, and foot fractures decreased by 39.0, 41.1, and 33.3%. Proximal tibial fractures increased both in absolute numbers and in incidence in the age groups 0–4, 10–14, and 15–19 years (IRR ≥ 1.1). The overall BGR was 2.3 in 2002 and 2.0 in 2017, indicating that the number of girls relative to that of boys who suffered a lower extremity fracture was higher in 2017 than in 2002. Furthermore, the BGR of all fracture locations increased with age in both years.

**Conclusions:**

The number of paediatric lower extremity fractures treated in German hospitals in 2017 was significantly lower than that in 2002. However, the fracture frequency in girls decreased to a lesser extent than that in boys. The incidence of proximal tibial fractures increased.

## Background

The risk of suffering a fracture is highest in childhood and in old age [1, 2]. However, fractures of the growing skeletal system have certain special features, especially with regard to demographic factors, accident mechanisms, and pathophysiology [3]. Certain paediatric fractures are particularly common in a certain age range. This is due not only to age-specific anatomical and physiological conditions (e.g., bone metabolism) but also to the activities of children (sports and leisure activities) [3–5]. In general, the peak age of paediatric patients with a fracture is approximately 14 years in boys and 12 years in girls, with boys being affected more frequently than girls [6, 7].

The treatment of orthopaedic injuries in children frequently occurs in hospitals and is associated with high costs for the health care system [8, 9]. Furthermore, paediatric fractures may be associated with temporary activity restrictions and long-term functional limitations, which may lead to a significant impairment of the children’s quality of life [10].

Upper extremity fractures are more common than lower extremity fractures in children, and the ratio is approximately 2 to 1 [3]. In the case of fractures resulting from falls, the fall characteristics and fracture locations vary with age. Whereas infants cannot brace falls, older children can increasingly brace falls with their extremities with increasing age. Therefore, long bone fractures after falls are most common in children aged 3 to 10 years [11].

Epidemiological studies have shown that the incidence of paediatric fractures changes over time. Several reasons, such as variations in prevalence of overweight, exposure to trauma, social environment, risk-taking behaviour, and ethnic composition of a population, have been discussed [6, 12–14]. Furthermore, the incidence and causes of paediatric fractures differ between different regions [15]. Variations in the living conditions of children – for example, new sports and leisure activities and participation in road traffic – may be associated with the variations in the incidence of paediatric fractures.

There are few recent epidemiological studies on the incidence of paediatric fractures in Germany [16]. A recent data analysis showed that the absolute number of paediatric upper extremity fractures treated in German hospitals was lower in 2017 than in 2002, whereas the incidence of in-hospital treatments of clavicle and forearm fractures was higher. The present study focuses on the incidence of lower extremity fractures [17]. Although lower extremity fractures are less common in children, the analysis of current fracture rates and epidemiological factors seems important because these fractures are associated with significant complications such as growth disturbances, deformities, early development of degenerative changes and associated health problems, and surgical interventions. Furthermore, the treatment concepts for paediatric lower extremity fractures have changed in recent decades [18]. The demand for a perfect result and shortened hospital stays has led to a trend towards operative treatment of paediatric fractures [19, 20].

The knowledge of current fracture rates and associated epidemiological factors is crucial for the adoption of targeted preventive measures and health care structures.

### Purpose

The primary aim of this study was to report fracture rates of paediatric lower extremity fractures treated in German hospitals in 2002 and 2017 and to detect changes over time.

The secondary aim was to evaluate the gender and age distribution for each fracture location.

### Hypothesis

We hypothesized that the incidence of different paediatric lower extremity fractures in 2002 and 2017 in German hospitals differed.

## Materials and methods

Data from the German National Hospital Discharge Registry, which is maintained by the German Federal Statistical Office and the Robert Koch Institute, were used for this study. This registry covers over 99% of all German hospitals and provides exact epidemiological data. Only data from inpatient treatments were included.

A registry query for the years 2002 and 2017 for the following fractures was performed according to the International Classification of Disease, German Modification (ICD-10-GM) codes [21, 22]:

Fractures of the hip and thigh: S72.0 femoral neck, S72.1 pertrochanteric femur, S72.2 subtrochanteric femur, S72.3 femoral shaft, S72.4 distal femur, S72.7 multiple fractures of the femur, S72.8 other parts of the femur, S72.9 femur, part unspecified. For the analysis of absolute fracture rates, per- (S72.1) and subtrochanteric femoral fractures (S72.2) were grouped together. Further, ‘S72.7 multiple fractures of the femur’, ‘S72.8 other parts of the femur’, and ‘S72.9 femur, part unspecified’ were grouped together as ‘Others’.

Fractures of the knee and lower leg: S82.0 patella, S82.1 proximal tibia, S82.2 tibial shaft, S82.3 distal tibia, S82.4 fibula alone, S82.5 medial malleolus, S82.6 lateral malleolus, S82.7 multiple fractures of the lower leg, S82.8 other parts of the lower leg, S82.9 lower leg, part unspecified. For the analysis of absolute fracture rates, ‘S82.7 multiple fractures of the lower leg’, ‘S82.8 other parts of the lower leg’ and ‘S82.9 lower leg, part unspecified’ were grouped together as ‘Others’.

Fractures of the foot: S92.0 calcaneus, S92.1 talus, S92.2 other tarsal bone(s), S92.3 metatarsal bone(s), S92.4 great toe, S92.5 other toes, S92.7 multiple fractures of the foot, S92.9 foot, unspecified. For the analysis of absolute fracture rates, ‘S92.4 great toe’ and ‘S92.5 other toes’ were grouped together as ‘Toes’.

The ICD-10 replaced the ICD-9 in 2000. To minimize documentation errors after changing to the ICD-10, the year 2002 was selected as the starting point for this analysis. Data from 2017 were the most recent data available.

The data obtained from the registry included the absolute frequencies as well as incidences of lower extremity fractures. Age at the time of fracture and gender were also captured. Patients up to 19 years of age were analysed, and the population was subdivided into four age groups: 0–4 years, 5–9 years, 10–14 years, and 15–19 years.

### Statistical analysis

The data were transferred into the software package Microsoft Excel (Microsoft Corp., Excel for Mac 2011, Version 14.7.7, Redmond, WA, USA). The boy: girl ratios (BGRs) for all fracture locations for the years 2002 and 2017 were calculated by dividing the absolute frequency of treated boys by that of treated girls in the same year. The fracture incidence in 2017 was compared with 2002 by calculating the incidence rate ratio (IRR).

### Compliance with ethical standards

For this study, anonymous data from the German National Hospital Discharge Registry were used. The study was conducted in agreement with the ethical standards of the institutional and national research committee and with the 1964 Helsinki declaration and its later amendments.

This is an epidemiological study with anonymized, centrally collected, and publicly available online data. No patient consent was required.

## Results

The Tables 1, 2, and 3 present the absolute fracture rates for 2002 and the percent change in fracture rates in 2017 for each fracture location – by age group as well as overall – for hip and thigh fractures, knee and lower leg fractures, and foot fractures. The BGRs are also presented for both years. The total number of fractures decreased from 28,808 in 2002 to 17,312 in 2017, which represents a decrease of 39.9%. The decrease in the incidence of hip and thigh fractures, knee and lower leg fractures, and foot fractures was 39.0, 41.1, and 33.3%, respectively.

**Table 1:** Hip and thigh fractures

	0–4 years				5–9 years				10–14 years				15–19 years				Overall			
	2002		2017		2002		2017		2002		2017		2002		2017		2002		2017	
	n	BGR	Δ (%)	BGR	n	BGR	Δ (%)	BGR	n	BGR	Δ (%)	BGR	n	BGR	Δ (%)	BGR	n	BGR	Δ (%)	BGR
Femoral neck (S72.0)	13	0.6	−23.1	2.3	47	1.9	−40.4	1.3	150	1.2	−30.0	1.9	112	2.9	−19.6	9.0	322	1.6	−27.6	2.9
Per- and subtrochanteric (S72.1 + S72.2)	83	1.5	−12.0	1.0	63	1.7	−27.0	1.1	98	3.3	−30.6	3.0	130	4.0	−41.5	4.4	374	2.6	−29.7	2.0
Femoral shaft (S72.3)	906	2.6	+4.6	2.6	690	2.0	− 37.5	2.7	457	2.2	−33.0	2.3	1041	4.1	−36.8	2.9	3094	2.8	−24.3	2.6
Distal femur (S72.4)	157	1.2	−30.6	0.9	165	1.3	−32.1	0.9	356	2.3	−34.3	2.0	500	4.3	−40.0	4.0	1178	2.4	−35.9	2.0
Others (S72.2 + S72.8 + S72.9)	348	1.9	−89.7	3.0	204	1.7	−95.1	1.5	181	2.0	−92.3	3.7	316	3.2	−95.6	1.8	1049	2.2	−92.9	2.5

Absolute fracture rates for 2002 and percent change in 2017 as well as BGRs for both years, by age group and overall. Per- (S72.1) and subtrochanteric femoral fractures (S72.2) were grouped together. Groups ‘S72.7 multiple fractures of the femur’, ‘S72.8 other parts of the femur’, and ‘S72.9 femur, part unspecified’ were grouped together as ‘Others’. *BGR* Boy: girl ratio

**Table 2:** Knee and lower leg fractures

	0–4 years				5–9 years				10–14 years				15–19 years				Overall			
	2002		2017		2002		2017		2002		2017		2002		2017		2002		2017	
	n	BGR	Δ (%)	BGR	n	BGR	Δ (%)	BGR	n	BGR	Δ (%)	BGR	n	BGR	Δ (%)	BGR	n	BGR	Δ (%)	BGR
Patella (S82.0)	1	0.0	−100.0		35	1.2	−74.3	0.5	326	1.8	−51.5	1.9	622	3.7	−53.5	3.5	984	2.7	−53.7	2.7
Proximal tibia (S81.1)	66	2.0	+74.2	1.1	151	1.2	−15.9	0.7	513	2.2	+15.0	3.8	718	3.5	+1.4	4.2	1448	2.5	+7.7	3.0
Tibial shaft (S82.2)	433	1.9	−19.4	1.3	975	2.0	−21.5	1.6	1490	2.0	−41.9	2.2	1445	5.7	−39.6	4.0	4343	2.7	−34.3	2.2
Distal tibia (S82.3)	267	1.2	−0.4	0.8	551	1.3	−21.8	1.0	2898	1.3	−38.1	1.1	1222	6.6	−42.6	5.3	4938	1.8	−35.3	1.4
Fibula alone (S82.4)	12	0.5	+25.0	1.5	25	0.9	−48.0	0.9	130	2.6	−76.9	1.1	196	3.1	−57.7	4.2	363	2.5	− 61.2	2.3
Medial malleolus (S82.5)	2	0.0	−50.0		43	1.2	−2.3	0.6	456	1.3	−39.7	0.9	717	3.1	−49.4	2.2	1218	2.1	−44.1	1.4
Lateral malleolus (S82.6)	7	0.8	−14.3	0.5	57	0.6	−52.6	0.5	450	1.2	−56.9	0.9	2447	3.1	−37.7	2.3	2961	2.5	−40.9	1.9
Others (S82.7 + S82.8 + S82.9)	158	2.1	−87.3	2.3	421	1.6	−89.3	1.0	1440	1.7	−78.3	1.1	1634	2.9	−56.8	1.5	3653	2.2	−70.3	1.4

Absolute fracture rates for 2002 and percent change in 2017 as well as BGRs for both years, by age group and overall. Groups ‘S82.7 multiple fractures of the lower leg’, ‘S82.8 other parts of the lower leg’ and ‘S82.9 lower leg, part unspecified’ were grouped together as ‘Others’. Missing data regarding gender: Group distal tibia, 2017, 10–14 years, *n* = 1; Group lateral malleolus, 2017, 15–19 years, *n* = 1. *BGR* Boy: girl ratio

**Table 3:** Foot fractures

	0–4 years				5–9 years				10–14 years				15–19 years				Overall			
	2002		2017		2002		2017		2002		2017		2002		2017		2002		2017	
	n	BGR	Δ (%)	BGR	n	BGR	Δ (%)	BGR	n	BGR	Δ (%)	BGR	n	BGR	Δ (%)	BGR	n	BGR	Δ (%)	BGR
Calcaneus (S92.0)	9	1.3	−44.4	0.7	45	2.5	−40.0	5.8	63	4.7	+4.8	2.7	167	3.3	−38.3	3.5	284	3.2	−29.2	3.2
Talus (S92.1)	5	4.0	−20.0	3.0	22	1.4	−45.5	1.0	73	1.1	−35.6	1.0	241	1.5	−35.7	1.8	341	1.4	−36.1	1.5
Other tarsal bone(s) (S92.2)	0			0.0	7	1.3	+42.9	0.7	47	2.6	−21.3	1.1	97	2.1	+9.3	2.3	151	2.2	+2.6	1.7
Metatarsal bone(s) (S92.3)	36	3.5	+8.3	1.3	104	1.5	−9.6	1.8	525	1.9	−43.0	1.5	740	2.6	−24.6	2.1	1405	2.2	−29.5	1.8
Toes (S92.4 + S92.5)	26	1.6	+11.5	1.9	59	0.9	−6.8	1.2	211	2.1	−39.8	1.6	300	4.1	−54.7	4.9	596	2.5	−41.8	2.2
Multiple (S92.7)	4	3.0	−100.0		8	0.6	−87.5		16	2.2	−87.5	0.0	36	4.1	−80.6		64	2.6	−84.4	4.0
Unspecified (S92.9)	3	0.5	−100.0		6	2.0	−100.0		12	3.0	−100.0		21	3.2	− 85.7	0.5	42	2.5	−92.9	0.5

Absolute fracture rates for 2002 and percent change in 2017 as well as BGRs for both years, by age group and overall. Groups ‘S92.4 great toe’ and ‘S92.5 other toes’ were grouped together as ‘Toes’. *BGR* Boy: girl ratio

There was a decrease in absolute fracture rates in all 20 subgroups analysed except the two subgroups of proximal tibial fractures (1448 fractures in 2002 versus 1560 fractures in 2017) and other tarsal fractures (151 fractures in 2002 versus 155 fractures in 2017). The incidence of proximal tibial fractures increased markedly in the group of 0–4-year-olds and to a lesser extent in the groups of 10–14- and 15–19-year-olds. The most common fracture locations in 2002 were femoral shaft, tibial shaft, distal tibia, and lateral and medial malleolus. The absolute number of all these fractures was lower in 2017 than in 2002 in all age groups.

The overall BGR was 2.3 in 2002 and 2.0 in 2017, which indicates that the number of girls relative to that of boys who suffered a lower extremity fracture was higher in 2017 than in 2002. All age groups showed a lower BGR in 2017 than in 2002 (0–4 years: BGR 1.9 to 1.6; 5–9 years: 1.6 to 1.4; 10–14 years: 1.7 to 1.4; 15–19 years: 3.6 to 2.9). Taking all age groups together, every fracture location was more common in boys than in girls in 2002 (BGR ≥ 1.4). This phenomenon was also observed in 2017 (BGR ≥ 1.4), except for the subgroup of ‘unspecified’ foot fractures, which was a very small group (*n* = 3, BGR = 0.5). In the groups of 0–4-year-olds and 5–9-year-olds, fractures in some locations occurred more frequently in girls than in boys in both years, whereas in the 10–14-year-olds, this was the case in only two fracture locations in 2017 (medial malleolus: BGR = 0.9; lateral malleolus BGR = 0.9). Furthermore, in the group of 15–19-year-olds, all fractures were more frequent in boys than in girls in both years (BGR ≥ 1.5) – except in the 2017 ‘unspecified’ foot fracture subgroup. Furthermore, the BGR for all fracture locations increased with age in both years.

**Fig. 1:**
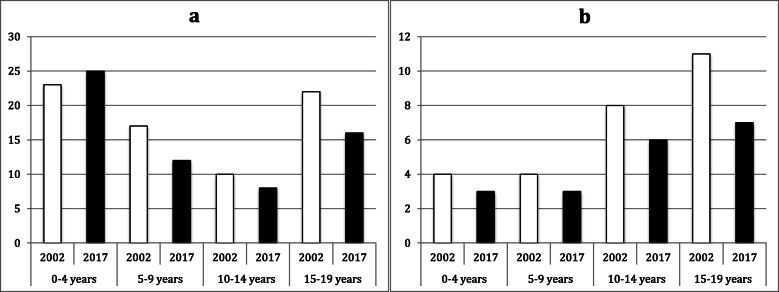
Fracture incidence per 10^5^ person-years by age group and year for (**a**) femoral shaft (S72.3) and (**b**) distal femoral fractures (S72.4)

Table 4 shows the fracture incidence by age group in 2002 and 2017 as well as the IRRs in each group. Among hip and thigh fractures, femoral shaft and distal femoral fractures had the highest incidence in both years. In the group of 0–4-year-olds, the incidence of femoral shaft fractures was slightly higher in 2017 than in 2002 (IRR = 1.1), whereas the incidence of distal femoral fractures was lower in 2017 than in 2002. In the remaining three age groups, the incidence of these two fractures was lower in 2017 than in 2002 (IRR ≤ 0.8; see Fig. 1).

**Table 4:** Fracture incidence in 2002 and 2017 (per 10^5^ person-years)

		0–4 years			5–9 years			10–14 years			15–19 years		
		2002	2017	IRR	2002	2017	IRR	2002	2017	IRR	2002	2017	IRR
**Hip and thigh**	Femoral neck (S72.0)	0	0		1	1	1.0	3	3	1.0	2	2	1.0
	Pertrochanteric (S72.1)	0	0		1	0	0.0	1	1	1.0	1	1	1.0
	Subtrochanteric (S72.2)	2	2	1.0	1	1	1.0	1	1	1.0	1	1	1.0
	Femoral shaft (S72.3)	23	25	1.1	17	12	0.7	10	8	0.8	22	16	0.7
	Distal femur (S72.4)	4	3	0.8	4	3	0.8	8	6	0.8	11	7	0.6
	Multiple fractures of the femur (S72.7)	0	0		0	0		0	0		0	0	
	Other parts of the femur (S72.8)	0	0		0	0		0	0		0	0	
	Femur, part unspecified (S72.9)	9	1	0.1	5	0	0.0	4	0	0.0	6	0	0.0
**Knee and lower leg**	Patella (S82.0)	0	0		1	0	0.0	7	4	0.6	13	7	0.5
	Proximal tibia (S81.1)	2	3	1.5	4	3	0.8	11	16	1.5	15	17	1.1
	Tibial shaft (S82.2)	11	9	0.8	24	21	0.9	32	23	0.7	31	21	0.7
	Distal tibia (S82.3)	7	7	1.0	14	12	0.9	62	48	0.8	26	17	0.7
	Fibula alone (S82.4)	0	0		1	0	0.0	3	1	0.3	4	2	0.5
	Medial malleolus (S82.5)	0	0		1	1	1.0	10	7	0.7	15	9	0.6
	Lateral malleolus (S82.6)	0	0		1	1	1.0	10	5	0.5	52	37	0.7
	Multiple fractures of the lower leg (S82.7)	1	0	0.0	3	0	0.0	5	0	0.0	5	0	0.0
	Other parts of the lower leg (S82.8)	0	0		2	1	0.5	16	8	0.5	21	16	0.8
	Lower leg, part unspecified (S82.9)	3	0	0.0	5	0	0.0	10	1	0.1	9	1	0.1
**Foot**	Calcaneus (S92.0)	0	0		1	1	1.0	1	2	2.0	4	2	0.5
	Talus (S92.1)	0	0		1	0	0.0	2	1	0.5	5	4	0.8
	Other tarsal bone(s) (S92.2)	0	0		0	0		1	1	1.0	2	3	1.5
	Metatarsal bone(s) (S92.3)	1	1	1.0	3	3	1.0	11	8	0.7	16	13	0.8
	Great toe (S92.4)	0	0		1	1	1.0	3	2	0.7	4	2	0.5
	Other toes (S92.5)	0	0		1	1	1.0	2	1	0.5	3	1	0.3
	Multiple (S92.7)	0	0		0	0		0	0		1	0	0.0
	Unspecified (S92.9)	0	0		0	0		0	0		0	0	

*IRR* Incidence rate ratio

**Fig. 2:**
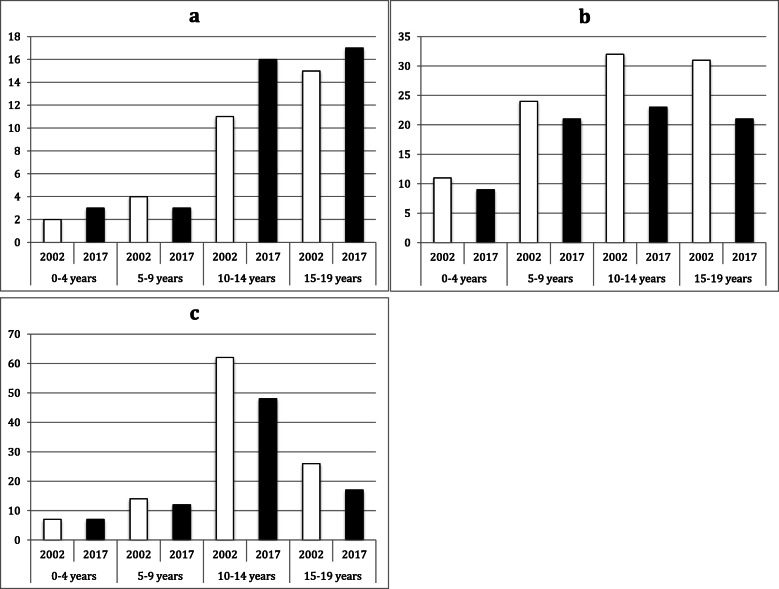
Fracture incidence per 10^5^ person-years by age group and year for (**a**) proximal tibia (S81.1), (**b**) tibial shaft (S81.2), and (**c**) distal tibial fractures (S81.3)

**Fig. 3:**
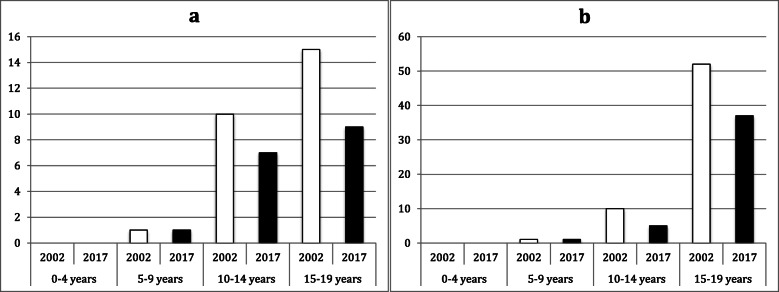
Fracture incidence per 10^5^ person-years by age group and year for (**a**) medial (S82.5) and (**b**) lateral malleolar fractures (S82.6)

Among knee and lower leg fractures, tibial and malleolar fractures had the highest incidence. Proximal tibial fractures were most common in the age groups 10–14 years and 15–19 years, showing a higher incidence in 2017 than in 2002 in both age groups (10–14 years: IRR = 1.5; 15–19 years; IRR 1.1; see Fig. 2). Both tibial shaft and distal tibial fractures showed peak incidence in the group of 10–14-year-olds. The incidence of these fractures was lower in all groups in 2017 than in 2002 (IRR ≤ 0.9), with the exception of distal tibial fractures in the group of 0–4-year-olds, where the incidence remained the same (IRR = 1.0). Malleolar fractures occurred in the age group of 10–14-year-olds but showed highest incidence in the age group 15–19-year-olds; the incidence of medial and lateral malleolar fractures was lower in both age groups in 2017 than in 2002 (IRR ≤ 0.7; see Fig. 3). The group of fractures of ‘other parts of the lower leg (S82.8)’ had the highest incidence between 10 and 19 years with a decreasing fracture incidence from 2002 to 2017 (10–14 years: IRR = 0.5; 15–19 years: IRR = 0.8).

**Fig. 4:**
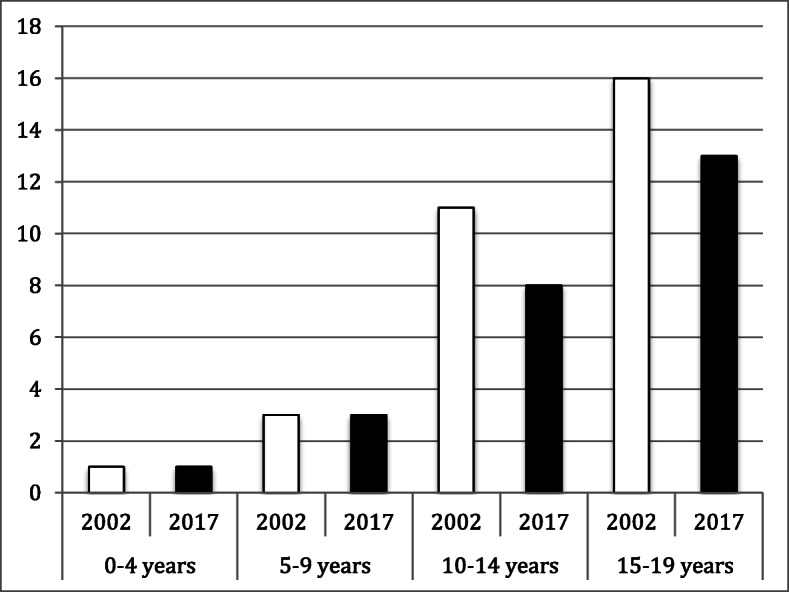
Fracture incidence per 10^5^ person-years by age group and year for metatarsal fractures (S92.3)

The incidence of foot fractures was lower than that of hip and thigh fractures and knee and lower leg fractures. Within the group of foot fractures, metatarsal fractures were the most common with the highest incidence in the two older age groups. In both these age groups, the incidence was lower in 2017 than in 2002 (10–14 years: IRR = 0.7; 15–19 years: IRR = 0.8; see Fig. 4).

**Fig. 5:**
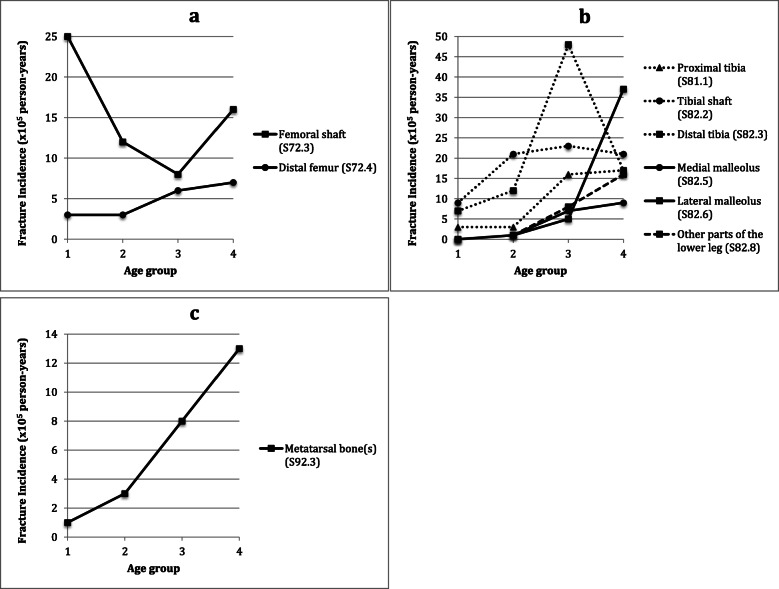
Fracture incidence of nine most common lower extremity fractures by age group in 2017. **a** hip and thigh, **b** knee and lower leg, and (**c**) foot. 1, 0–4 years; 2, 5–9 years; 3, 10–14 years; 4, 15–19 years

Figure 5 shows the age distribution pattern of the nine most common fracture location based on the incidence values for 2017.

## Discussion

Regarding the primary aim of our study, we found that the absolute number of paediatric lower extremity fractures treated in German hospitals in 2017 was lower by 39.9% than that in 2002. The results must be interpreted against the background of a demographic change in the population between 2002 and 2017. The group of individuals under 20 years of age consisted of approximately 4.6 million individuals in 2002 and approximately 4.1 million individuals in 2017. This reflects a general demographic trend of a decrease and increase, respectively, in the number of young and old individuals in Germany [23].

Femoral shaft, tibial shaft, distal tibia, and lateral/medial malleolus remained the most common fracture locations in 2017. Nevertheless, the most important finding regarding time trends was that the number of proximal tibial fractures in the age groups 10–14 years and 15–19 years was higher in 2017 than in 2002. However, proximal tibial fractures accounted for a small proportion of all knee and lower leg fractures, whereas tibial shaft and distal tibial fractures were the most common in this group in both years. Nevertheless, the proportion of proximal tibial fractures in this group increased from 7.3% in 2002 to 13.3% in 2017. The proportion of proximal tibial fractures in total lower extremity fractures increased from 5.0% in 2002 to 9.0% in 2017. Proximal tibial fractures are particularly associated with sports accidents and are usually the result of indirect force [24]. Proximal tibial physeal fractures mainly affect children between the ages of 12 and 14 years. They are associated with ligamentous injuries in approximately 40% of cases; the risk of concomitant ligamentous injury increases with increasing patient age. Furthermore, there is an association with concomitant neurovascular injuries in approximately 14% of cases and an approximately 25% risk of posttraumatic growth disturbances. Physeal injury can lead to asymmetric physeal closure or complete physeal closure owing to physeal bar formation. This may result in growth disturbances such as leg length discrepancy or angular deformity. Metaphyseal proximal tibial fractures are most common between the ages 3 and 6 years; these fractures are associated with a high rate of posttraumatic genu valgum [24]. In summary, paediatric proximal tibial fractures have a high complication rate. We believe that the increase in proximal tibial fractures is also owing to a higher detection rate because of the increased use of computed tomography and magnetic resonance imaging [25].

Data from epidemiological studies on paediatric fractures are difficult to compare because of different study design; the data sources, periods of time under study, and age groups of the examined patients are different. In addition, only a few studies have investigated time trends. These factors make it difficult to compare our findings with existing studies. A Finnish study investigated the epidemiology of paediatric fractures in Helsinki in 2005 [26]. The findings were compared to older available data, which included in- and outpatient treatments. They reported an annual fracture incidence of 163 per 10,000 for children between 0 to 15 years. They also reported that 22% of 1396 fractures affected the lower extremities. Fractures of the metatarsals, toes, and malleoli were the most common lower extremity fractures with rates of 5.1, 4.4, and 4.3% of all fractures, respectively. By comparing data from 2005 with older data from 1983 for children between 0 to 14 years, they identified a decrease in the incidence of lower extremity fractures by 34% [26]. The authors believed that improved living standards and education levels, decreased number of siblings in families, mass movements from rural areas to cities, and improved traffic and playground safety were the possible explanations for these findings. Furthermore, they reported that Finnish children are more physically active; however, the physical activity of teenagers has declined, and the rate of overweight in this group has increased [26]. Our study also examined older children and adolescents up to 19 years of age and covered a more recent time period, but in terms of time trends of lower extremity fracture frequencies, our data point in the same direction although the most common fracture locations in our study population differed.

Another study investigating time trends comes from a hospital in northern Sweden [13]. Between 1993 and 2007, an increase of 13% in fractures was observed in patients under 20 years of age. Among lower extremity fractures, ankle, toe, and metatarsal fractures were the most common, similar to the findings of the Finnish study. The authors speculated that a change in children’s activity pattern was responsible for the increase. The difference in fracture frequencies between our results and the studies cited may be explained by the fact that our data include only hospitalised patients.

Another Swedish study investigated the fracture incidence of children under 16 years old in Malmö [6]. They compared data from 2005/2006 with older data from 1993/1994 and found a decrease in the overall fracture rates in girls but not in boys. Differences in the prevalence of overweight, exposure to trauma, social environment, risk-taking behaviour, and physical activity levels; improvements in traffic safety; and structured paediatric injury prevention in the home environment as well as the distribution of ethnicity in a population were suggested to be the reasons for the time trends in fracture rates as well as differences between different geographic regions [6].

The analysis of accident type is important for the analysis of time trends in the frequency of certain fractures. Falls and accidents during leisure activities are the most common accident types [27]. However, there is also age- and gender-specific variability [27, 28]. Certain extreme sports (e.g., skateboarding, snowboarding, and mountain biking) are increasingly popular among children and are associated with an increased fracture risk [3], particularly affecting school children and adolescents, and fractures often occur during the phase of learning the new activity [3]. German data from 2003 indicate that the majority of paediatric long bone fractures resulted from accidents in sports (38.5%), at home (23.0%), and on playgrounds (19.9%) [16].

An increasing number of children are affected by obesity [29]. Obesity in childhood is associated with an increased fracture risk [30]. Fat has a negative impact on the skeleton, either directly or by reduced gain in bone mass in relation to body size [30]. Furthermore, the higher risk of fractures is the result of a greater risk of falls owing to poor balance and gait abnormalities, greater force generation upon impact, and unhealthy diet with insufficient calcium intake [30]. Leisure activities with low physical activity and high screen time also seem to increase the risk of fractures rather than protect against them [18]. Current data confirm that vitamin D deficiency in children occurs in Western European countries [31]. But there is disagreement as to whether reduced vitamin D levels (except for rickets) increase the risk of fractures in children [32].

The secondary aim of our study was to evaluate the gender and age distribution for each fracture location. The overall BGR was 2.3 in 2002 and 2.0 in 2017, and the BGR increased with age in both years. This is particularly remarkable against the background that, in the four age groups, boys were represented more frequently than girls in both years. The population data of Germany from 2002 and 2016 indicate that the proportion of boys in the four age groups is 51.3–51.4% for 2002 and 51.4–52.7% for 2016 [33]. Data from 2017 were not yet available. One reason for the relative increase in the incidence of fractures in girls may be that girls are increasingly engaged in risky leisure activities. The BGRs reported in previous studies range between 1.5 and 5.5 [6, 13, 26, 27, 34]; the wide distribution is most likely to be explained by the different composition of the study populations.

Regarding the age distribution for the different fractures, our analysis confirms known characteristics. Our findings demonstrate that infants and preschool children predominantly suffer fractures of the shafts of the long bones (femur and tibia), and the risk of fractures with potential joint involvement (distal femur, proximal and distal tibia, and ankle) increases with age. This phenomenon is found in both 2002 and 2017 data. In this respect, our results agree with those of an older analysis of paediatric fractures from Germany, Austria, and Switzerland in 2003 [16].

A limitation of our study is that it includes data from only hospitalised patients, and outpatients are not included. The proportion of patients who undergo surgical treatment may be higher in the group of hospitalised patients than in the group of patients treated in an outpatient setting, which represents a selection bias. However, because lower extremity fractures often lead to an inability to walk, it can be assumed that a large proportion of patients are treated as inpatients. In this respect, the total incidence of all paediatric lower extremity fractures in Germany cannot be established. Because our evaluation is based on the ICD-10, we cannot make any statements about the exact fracture patterns. The German National Hospital Discharge Registry only uses the first three digits of the ICD-10 code. Therefore, we could classify fractures only according to bone and bone segment. Different fractures were grouped together based on the anatomical location (e.g., physeal and metaphyseal proximal tibial fractures grouped together in the S81.1 group). Because evaluation was possible only on the basis of the four age groups, more precise conclusions about age distribution could not be drawn.

The greatest strength of our study is that it presents Germany-wide data from almost all hospitals. We assume that our results are also valid to a certain extent for other countries with highly developed health care systems. In addition, by comparing the data from 2002 and 2017, we could analyse the time trends in fracture rate, incidence, and demographics.

Our results can be used to specifically optimise prevention strategies. Preventive measures should be directed, in particular, against obesity in children because being overweight increases the risk of fractures [35]. In addition, overweight in children leads to a smaller increase in bone mass and ultimately to a lower peak bone mass, which in turn is associated with a higher risk of fracture in later life [35]. In this context, girls, in particular, should be focused on because the fracture frequency in girls decreased to a lesser extent than that in boys. The reasons for this finding (e.g., participation in riskier sports and leisure activities) should be investigated in future studies. The most common lower extremity fracture locations in paediatric patients treated in hospitals remained femoral shaft, tibial shaft, distal tibia, and lateral/medial malleolus, but their incidence was lower in 2017 than in 2002. However, the treatment concepts for these fractures should be focused on because these fractures are associated with a high complication rate [19]. In addition, the treatment of proximal tibial fractures should also be examined as this specific injury becomes more common.

## Conclusions

The number of paediatric lower extremity fractures treated in German hospitals was significantly lower in 2017 than in 2002, which reflects a demographic change with a decrease and increase, respectively, in the number of young and old individuals. The fracture frequency in girls decreased to a lesser extent than that in boys. Preventive measures should therefore be particularly targeted at girls. While the frequency of all significant lower extremity fractures decreased, the incidence of proximal tibial fractures increased. Further research about accident types associated with these fractures and their clinical management should be carried out to initiate preventive measures and improve treatment.

## Data Availability

The datasets analysed in the current study are online publicly available: http://www.gbe.bund.de.

## Abbreviations

BGR: Boy: girl ratio
IRR: Incidence rate ratio
ICD-10-GM: International Classification of Disease, German Modification
